# Biopsy of distant metastasis is not a significant prognostic factor for synchronous metastatic nasopharyngeal carcinoma: a propensity score-matched analysis from the Surveillance Epidemiology and End-Results Registry

**DOI:** 10.7150/jca.54686

**Published:** 2021-05-27

**Authors:** Mei Lin, Qi Yang, Xiong Zou, Rui You, Chong-Yang Duan, You-Ping Liu, Pei-Yu Huang, Yu-Long Xie, Zhi-Qiang Wang, Ting Liu, Si-Yuan Chen, Yi-Jun Hua, Ming-Yuan Chen

**Affiliations:** 1 Department of Nasopharyngeal Carcinoma, Sun Yat-sen University Cancer Center, 651 Dongfeng East Road, Guangzhou 510060, P. R. China.; 2 Sun Yat-sen University Cancer Center, State Key Laboratory of Oncology in South China, Collaborative Innovation Center for Cancer Medicine.; 3 Guangdong Key Laboratory of Nasopharyngeal Carcinoma Diagnosis and Therapy, Guangzhou 510060, China.; 4 Department of Biostatistics, School of Public Health, Southern Medical University, Guangzhou, 510515, China.

**Keywords:** nasopharyngeal carcinoma, distant metastasis, biopsy, prognostic factors

## Abstract

**Introduction:** Biopsy is essential for some patients with suspected distant metastasis, so we aim to figure out whether biopsy of distant metastasis is associated with impaired survival in NPC.

**Methods:** A total of 743 synchronous metastatic NPC patients from 2004 to 2016 were analyzed from the population-based Surveillance, Epidemiology, and End Results program. Propensity score matching was used to control confounders and create a well-balanced cohort. Five-year survival rate estimates and Kaplan-Meier survival curves were calculated. Cox proportional hazard ratios (HRs) were used to identify independent prognostic factors for survival.

**Results:** Of 743 eligible patients, 194 (26.11%) underwent biopsy of distant metastasis. After control for demographic and clinicopathologic characteristics, patients with biopsy of distant metastasis achieved comparable 5-year overall survival (OS) (20.3% vs 24.7%; P = 0.41) and 5-year cancer specific survival (CSS) (31.0% vs 33.6%; P = 0.35) with patients without biopsies. Multivariate analysis further confirmed that biopsy of distant metastasis was not associated with impaired OS (HR = 1.03, 95% CI = 0.84-1.25; P = 0.80) or CSS (HR = 1.07, 95% CI = 0.86-1.34; P = 0.54).

**Conclusions:** Biopsy of distant metastasis was not associated with impaired survival outcomes for synchronous metastatic NPC patients. Biopsy of distant metastasis could be another diagnosed choice for patients with suspected distant metastasis.

## Introduction

Nasopharyngeal carcinoma (NPC) is prevalent in southeast Asia and northern Africa, with an age-standardized rate of 3.0 per 100,000 in China to 0.4 per 100,000 in population that are mainly white 1, 2. There are about 5% NPC patients develop distant metastasis at diagnosis, which is also called synchronous metastatic NPC 3, and 20~30% patient suffer from metastasis after standard chemoradiotherapy 4-7. Unfortunately, median overall survival (OS) time of patients with distant metastasis is as short as 10-20 months 8-10. Distant metastasis accounts for about 70% of treatment failures in NPC.

Conventionally, the diagnosis of metastasis counts on non-invasive modalities like magnetic resonance imaging (MRI), computed tomography (CT) and [18F] fluorodeoxyglucose positron emission tomography and CT (PET-CT) 11. Although the precision of diagnosis of distant metastasis is high using non-invasive patients, there are still patients with ambiguous distant metastasis who might receive biopsy of suspected metastatic sites. The application of novel targeted therapies like immune checkpoint inhibitors (ICIs) for metastatic NPC patients mainly depends on the expression of immune checkpoint molecules in primary tumor 12. With the advent of precision medicine, whether the molecular characteristics of primary tumor can represent that of metastatic lesions and provide enough guidance of targeted therapies concerns many clinicians. It has been reported that genetic characteristics of distant metastasis were distinctly different from that of primary tumors, based on results of high-throughput sequencing on paired primary and distant metastatic tumors 13-15. Peter J. Campbell et al. found that most distant metastases acquired driver mutations not seen in the primary breast tumor 15. To settle the problem whether microenvironment is distinctly different between primary tumor and distant metastasis, biopsy of metastatic sites is needed.

Concerns about promotion of cell dissemination impeded the application of biopsy of neck and metastatic sites in NPC patients 16, 17. Previous studies have shown that cervical lymph node biopsy was not associated with impaired survival in NPC 18, 19. And Fine-needle aspiration biopsy of the neck has been clearly recommended in work up of NPC in National Comprehensive Cancer Network (NCCN) guidelines in 2015 20. However, there is no information concerning the relationship between survival and biopsy of distant metastasis. In this study, we aimed to evaluate the association between biopsy of distant metastasis and survival based on a retrospective population-based study carried on data from the Surveillance, Epidemiology, and End Results Program (SEER).

## Methods

### Patient and study design

A retrospective cohort study was conducted with SEER incidence 18 Program of the National Cancer Institute 21 (Nov 2018 Submission). Data from states of Atlanta, Greater Georgia, Rural Georgia, San Francisco-Oakland, San Jose-Monterey, Greater California, Hawaii, Iowa, Kentucky, Los Angeles, Louisiana, New Mexico, New Jersey, Seattle-Puget Sound and Utah; and three supplemental registries (Alaska Native Tumor Registry, Connecticut and Detroit) were included in SEER incidence 18 Program. SEER*Stat v8.6.3.1 was used to access the data.

**Figure 1:**
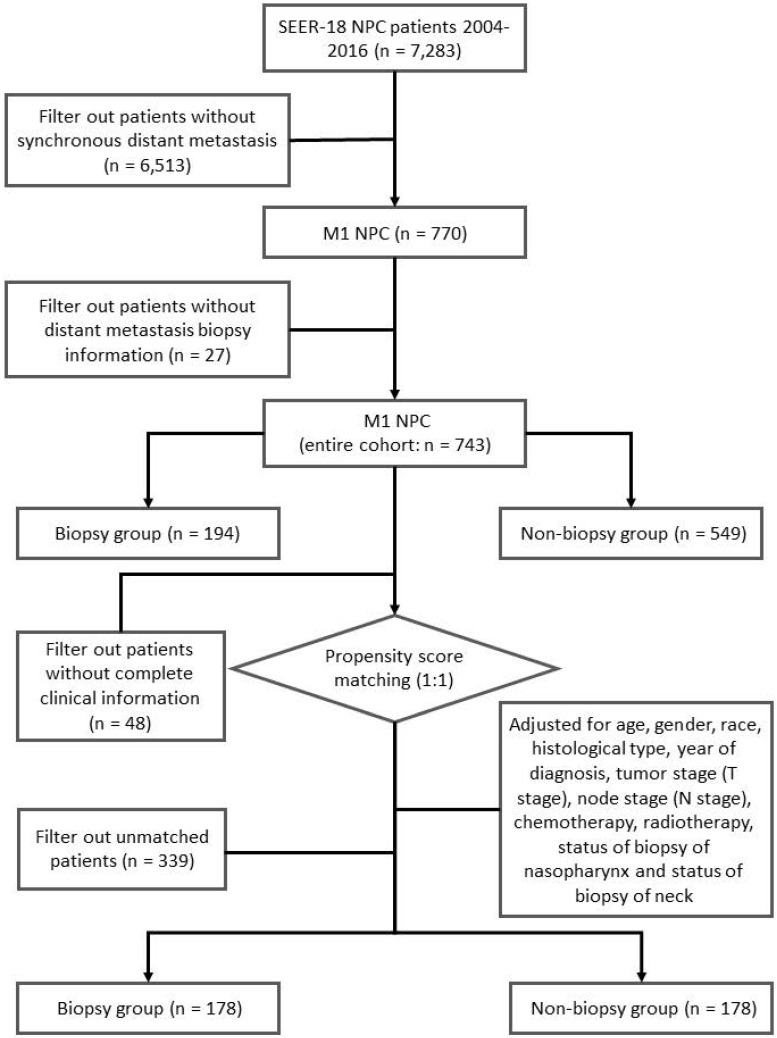
Flow chart of our study.

We identified 7,283 incident NPC cases from 2004 to 2016. Tumor site and histology were coded according to criteria specified by the World Health Organization (WHO) in International Classification of Diseases for Oncology (ICD-O-3) 22. For patients diagnosed between 2004 and 2015, TNM stage was identified using the American Joint Committee on Cancer (AJCC) 6^th^ stage. For patients diagnosed in 2016, SEER combined summary stage was used. All stages were converted to the AJCC 7^th^ stage. In our study, synchronous metastatic NPC patients were included. The status of biopsy of distant metastasis were identified using “CS Mets Eval (2004-2015)” and “Derived SEER Combined M src (2016+)” variables, patients without complete information of biopsy were excluded (**Figure 1**). A total of 743 synchronous metastatic NPC patients were analyzed in this study.

To control for confounding factors and minimize bias, two cohorts were generated via propensity score matching (PSM) 23. Patients with missing value were removed before PSM analysis. Adjusted for age, gender, race, histological type, year of diagnosis, tumor stage (T stage), node stage (N stage), chemotherapy, radiotherapy, status of biopsy of nasopharynx and status of biopsy of neck, a well-balanced cohort by matching each patient who underwent biopsy of distant metastasis with one patient who did not receive biopsy of distant metastasis was created (**Figure 1**).

### Variable selection

The primary outcome was OS which was defined as the duration from diagnosis of distant metastasis to death by any causes or the last follow-up. And the cancer-specific survival (CSS) was defined as the duration from the date of diagnosis until death due to NPC other than other causes.

Histological types of the tumors were grouped according to the WHO classification scheme using the ICD-O-3 codes. The keratinizing squamous cell carcinoma (KSCC) group consists of squamous cell carcinoma (ICD-O codes 8070 and 8071). The differentiated non-keratinizing carcinoma group (DNKC) consists of large- and small-cell non-keratinizing carcinoma (ICD-O codes 8072 and 8073). And the undifferentiated non-keratinizing carcinoma group (UNKC) consists of undifferentiated carcinoma, lymphoepithelial carcinoma and basaloid squamous cell carcinoma (ICD-O codes 8020, 8082, and 8083). Patients with histological type other than previously mentioned were classified as the other group. Race was coded as American Indian/Alaska Native/black, Asian or Pacific Islander and white people. The status of biopsy of nasopharynx was identified under variables “CS Tumor Size-Ext Eval (2004-2015)” and “Derived SEER Combined T src-2016+”. And the status of biopsy of neck was identified using “CS Reg Node Eval (2004-2015)” and “Derived SEER Combined N src (2016+)” variables.

### Statistical analysis

Comparisons of demographic, clinical and pathologic variables between patients with various categories were carried out using Chi-square statistic for nominal variables and Student's t-test for continuous variables. Survival curves were constructed using the Kaplan-Meier method and compared with the log-rank test. Univariate and Multivariate Cox regression analyses were used to determine factors significantly associated with survival and to calculate hazard ratios (HRs) and 95% confidence interval (CI). Missing value were removed in the univariate and multivariate analyses. All statistical analyses were conducted using R version 3.5.0. Statistical significance was assumed for a two-tailed P value <0.05.

## Results

### Patient characteristics

In all, we investigated 743 NPC patients with distant metastasis at diagnosis, including 194 (26.11%) underwent biopsy of distant metastasis and 549 (73.89%) did not (**Table 1**). Table 1 showed the baseline characteristics of the two groups. Except T stage, the demographic, clinicopathologic characteristics showed no difference between the biopsy group and the non-biopsy group. Patients with advanced T stage (T3-4) was prevalent in non-biopsy group (50.45%) compared to the biopsy group (37.11%).

Adjusted for age, gender, race, histological type, year of diagnosis, tumor stage (T stage), node stage (N stage), chemotherapy, radiotherapy, status of biopsy of nasopharynx and status of biopsy of neck, a well-balanced cohort containing 178 patients in the biopsy group and 178 patients in the non-biopsy group was created (**Table 1**).

### Impact of biopsy of distant metastasis on survival outcomes

**Figure 2:**
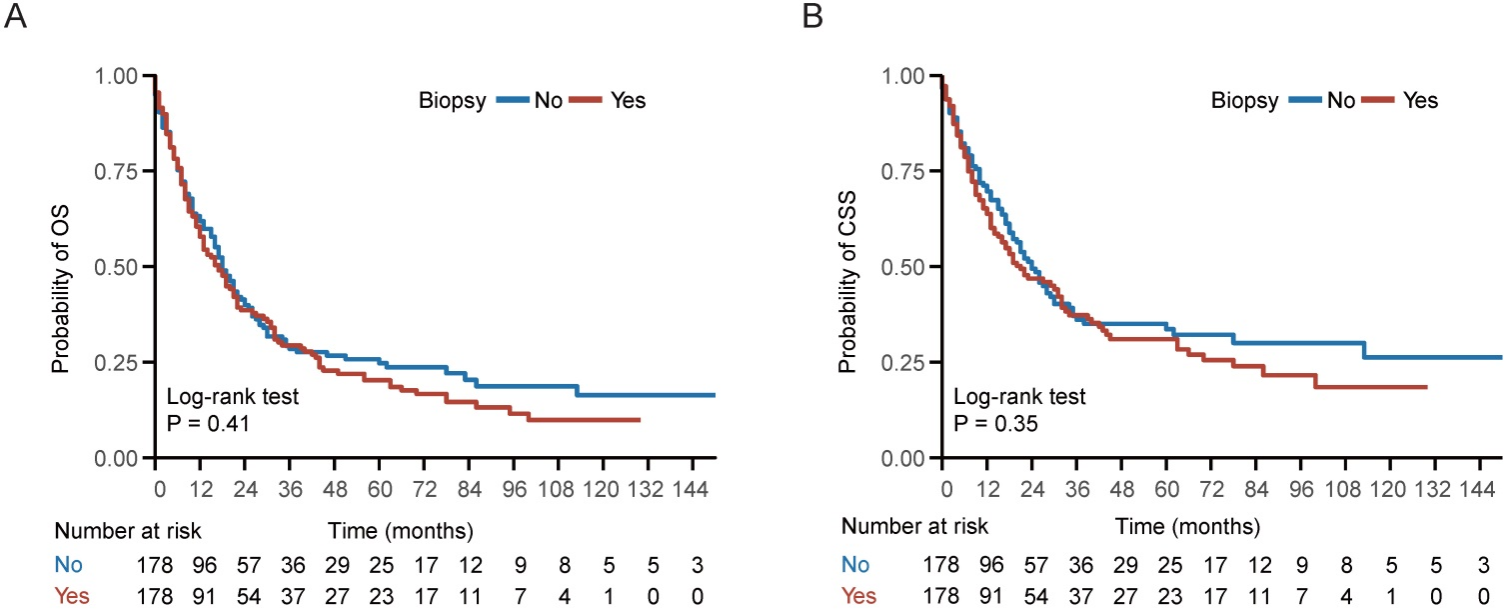
** Survival curve of patients with or without biopsy of distant metastasis.** (A-B) Kaplan-Meier (KM) curves of overall survival (OS) and cancer specific survival (CSS) of patients with or without biopsy of distant metastasis in the well-balanced cohort.

In the entire cohort, 534 (71.87%) patients died, including 424 (57.07%) patients die of NPC. Kaplan-Meier survival analysis demonstrated no statistically significant differences between the biopsy and non-biopsy groups in 5-year OS (20.3% vs 24.7%; P = 0.41) and 5-year CSS (31.0% vs 33.6%; P = 0.35) in the well-balanced cohort (**Figure 2**). Univariate analysis indicated that biopsy of distant metastasis was not associated with OS (HR = 1.11, 95% CI = 0.87-1.42; P = 0.41) or CSS (HR = 1.14, 95% CI = 0.86-1.52; P = 0.35) in the well-balanced cohort (**Table 2**). In addition, age > 60 years, American Indian/Alaska Native/black people and KSC histological type were associated with impaired OS and CSS (**Table 2**). After adjustment for age, gender, race, histological type, T stage, N stage, chemotherapy and radiotherapy status, multivariate Cox regression analysis indicated that biopsy of distant metastasis was not an independent prognostic factor for OS (HR = 1.03, 95% CI = 0.84-1.25; P = 0.80) or CSS (HR = 1.07, 95% CI = 0.86-1.34; P = 0.54) in the entire cohort (**Table 3**).

### Subgroup analysis

**Figure 3:**
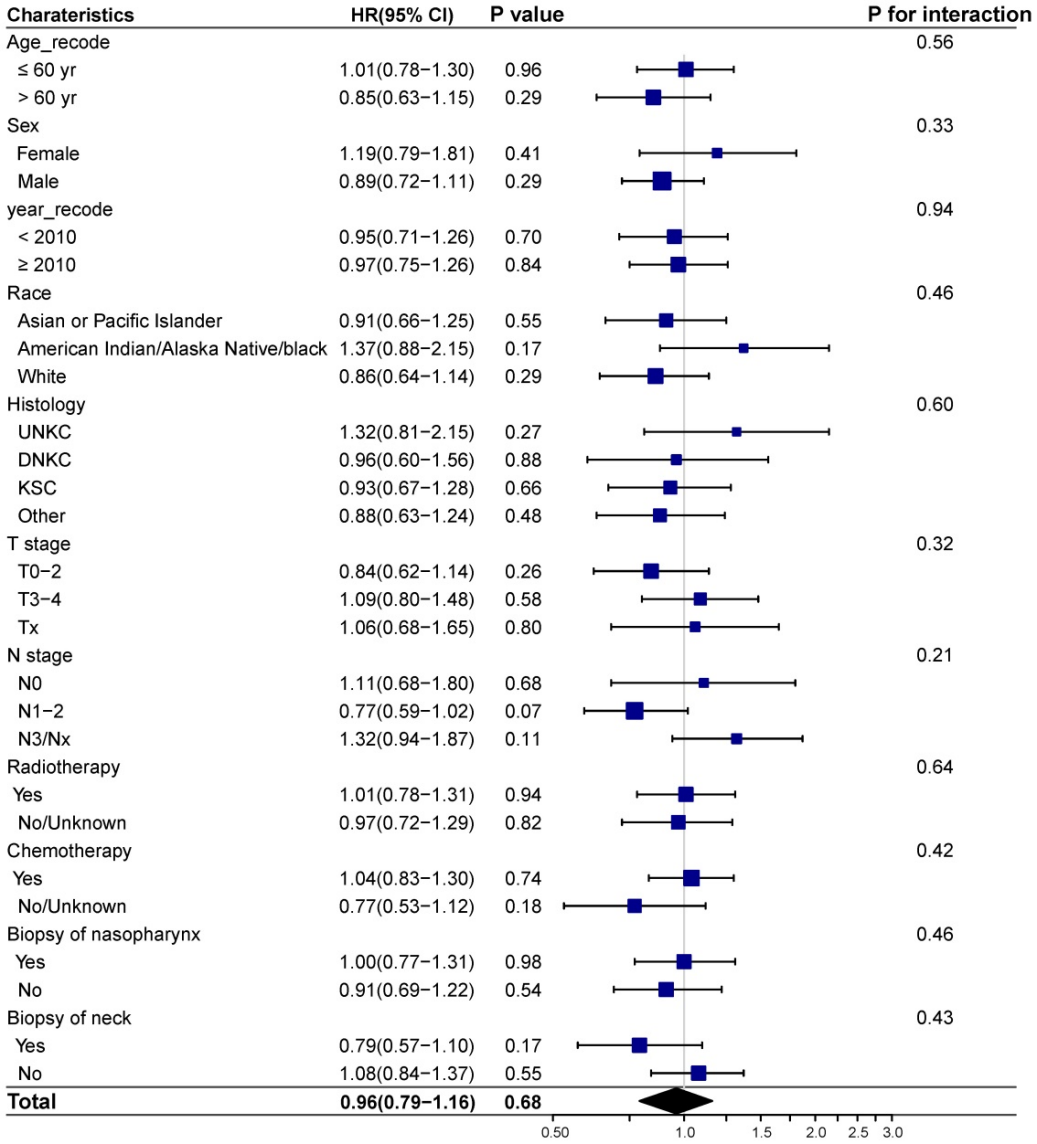
** Forest plot showing the relationship of biopsy of distant metastasis and overall survival in different subgroups.** Abbreviations: HR: Hazard ratio; CI: Confidence interval; UNKC: Undifferentiated nonkeratinizing carcinoma; DNKC: Differentiated nonkeratinizing carcinoma; KSCC: Keratinizing squamous cell carcinoma.

**Figure 4:**
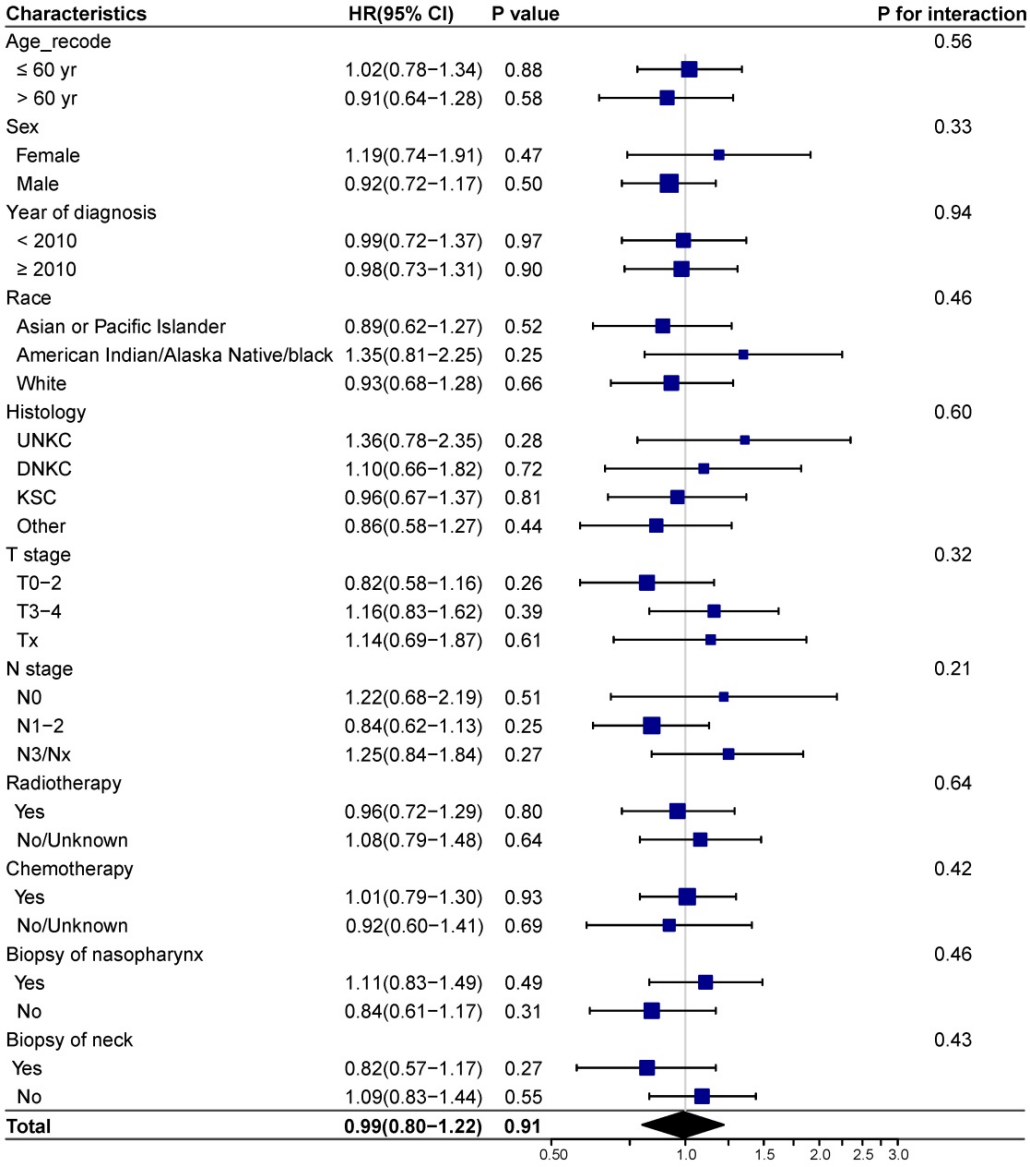
** Forest plot showing the relationship of biopsy of distant metastasis and cancer specific survival in different subgroups.** Abbreviations: HR: Hazard ratio; CI: Confidence interval; UNKC: Undifferentiated nonkeratinizing carcinoma; DNKC: Differentiated nonkeratinizing carcinoma; KSCC: Keratinizing squamous cell carcinoma.

We next investigated whether biopsy of distant metastasis is a prognostic factor for survival outcomes in different demographic and clinicopathologic subgroups based on the entire cohort. No interaction effect was detected between biopsy of distant metastasis and clinicopathological characteristics (**Figure 3-4**). And biopsy of distant metastasis was not associated with impaired OS or DSS in different subgroups (**Figure 3-4**).

## Discussion

Based on a well-balanced cohort, we for the first time found that synchronous metastatic NPC patients with biopsy of distant metastasis achieved comparable OS and CSS compared to those without biopsy. Biopsy of distant metastasis was not a significant prognostic factor for synchronous metastatic patients.

Biopsy is generally applied to obtain suspected tissues for histopathological diagnosis of cancer or other disease. With concern of the risk of tumor dissemination and discomfort that biopsy might bring about 4, 16, 24, scientists strived to establish the noninvasive methods for diagnosis like circulating tumor DNA 25, 26. However, biopsy of primary tumor remains cornerstone in most cancers. In breast cancer, the biopsy of clinically suspicious metastatic lesions could confirm diagnosis of metastases. Clemons, M. J. et.al also found that biopsy was well tolerated, and most patient reported reassurance at having tissue confirmation of metastatic disease 27. For some patients, it's hard to confirm distant metastasis merely depended on image data, and diagnostic therapies might be applied. Our study indicated that biopsy of distant metastasis was not associated with impaired OS and CSS in NPC, and might become another choice for those with suspected metastases.

It's worth noted that response rate of promising targeted therapies like vascular endothelial growth factor receptor inhibitors and ICIs in metastatic NPC remained as low as 30% 12, 28-31. The discordance of molecular characteristics between primary tumor and distant metastasis might contribute to the low efficacy rate of targeted therapy. Biopsy of distant metastasis might help to identify patients sensitive to targeted therapies. It's reported that biopsy of distant metastasis evaluated discrepancies between estrogen receptor, progesterone receptor and Her2 status and excluded secondary malignancy, which might change the therapeutic strategy and bring survival benefit for breast cancer patients 27, 32. Recently, we found that there were two different metastatic routes which showed distinct response rate for different therapy modalities in NPC patients by performing high-throughput sequencing on paired primary tumor, regional lymph nodes and distant metastasis samples (data unpublished). Thus, biopsy and targeted markers detection of distant metastasis might be conducive to decisions of therapies for NPC patients.

Our study has several limitations. Firstly, it is a retrospective study based on SEER database, some information like treatment information might be inaccurate. We excluded patients with incomplete information and conducted the subgroup analysis using OS and CSS as the survival outcomes to ensure the stability of our results. Secondly, it's reported that modality of biopsy and interval of receiving treatment after biopsy were associated with poor survival in NPC, but this information was not provided by the SEER program, and further research should take these factors into consideration. Thirdly, the compliance for biopsy of distant metastasis should be further explored in future studies. Finally, our findings need to be validated in a multi-institutional prospective study in the future.

In conclusion, biopsy of distant metastasis was not associated with impaired survival in synchronous metastatic NPC patients. Biopsy of distant metastasis could be recommended for patients with suspected distant metastasis or those showed resistance to chemoradiotherapy and might receive targeted therapies.

## Figures and Tables

**Table 1:** Comparison of baseline characteristics between the biopsy and non-biopsy groups before and after propensity score matching

Characteristics	Entire cohort	Biopsy of distant metastasis		*P* value^a^	Biopsy of distant metastasis		*P* value^a^
		Yes	No		Yes	No	
Total	743	194	549		178	178	
Age, median ± sd	56±16.6	56.5±16.7	56±16.6	0.4	57±15.4	59.5±16.6	0.98
**Sex**				0.87			0.90
Female, no. (%)	171 (23.01)	46 (23.71)	125 (22.77)		43 (24.16)	45 (25.28)	
Male, no. (%)	572 (76.99)	148 (76.29)	424 (77.23)		135 (75.84)	133 (74.72)	
**Year of diagnosis**				0.99			0.74
<2010, no. (%)	285 (38.36)	75 (38.66)	210 (38.25)		69 (38.76)	65 (36.52)	
≥2010, no. (%)	458 (61.64)	119 (61.34)	339 (61.75)		109 (61.24)	113 (63.48)	
**Race^ b^**				0.3			0.88
Asian or Pacific Islander, no. (%)	293 (39.43)	73 (37.63)	220 (40.07)		69 (38.76)	72 (40.45)	
American Indian/Alaska Native/black, no. (%)	128 (17.23)	28 (14.43)	100 (18.21)		26 (14.61)	23 (12.92)	
White, no. (%)	315 (42.40)	90 (46.39)	225 (40.98)		83 (46.63)	83 (46.63)	
**Histology**				0.67			1.00
UNKC, no. (%)	112 (15.07)	30 (15.46)	82 (14.94)		28 (15.73)	27 (15.17)	
DNKC, no. (%)	131 (17.63)	39 (20.10)	92 (16.76)		38 (21.35)	38 (21.35)	
KSC, no. (%)	247 (33.24)	59 (30.41)	188 (34.24)		56 (31.46)	58 (32.58)	
Other, no. (%)	253 (34.05)	66 (34.02)	187 (34.06)		56 (31.46)	55 (30.90)	
**T stage ^b^**				0			0.23
T0, no. (%)	9 (1.21)	8 (4.12)	1 (0.18)		8 (4.49)	1 (0.56)	
T1, no. (%)	180 (24.23)	49 (25.26)	131 (23.86)		48 (26.97)	58 (32.58)	
T2, no. (%)	80 (10.77)	22 (11.34)	58 (10.56)		22 (12.36)	23 (12.92)	
T3, no. (%)	145 (19.52)	28 (14.43)	117 (21.31)		25 (14.04)	28 (15.73)	
T4, no. (%)	204 (27.46)	44 (22.68)	160 (29.14)		43 (24.16)	39 (21.91)	
Tx, no. (%)	109 (14.67)	37 (19.07)	72 (13.11)		32 (17.98)	29 (16.29)	
**N stage ^b^**				0.54			0.98
N0, no. (%)	106 (14.27)	29 (14.95)	77 (14.03)		27 (15.17)	30 (16.85)	
N1, no. (%)	200 (26.92)	47 (24.23)	153 (27.87)		45 (25.28)	41 (23.03)	
N2, no. (%)	215 (28.94)	56 (28.87)	159 (28.96)		54 (30.34)	59 (33.15)	
N3a, no. (%)	23 (3.10)	5 (2.58)	18 (3.28)		5 (2.81)	6 (3.37)	
N3b, no. (%)	120 (16.15)	31 (15.98)	89 (16.21)		31 (17.42)	27 (15.17)	
N3, NOS, no. (%)	25 (3.36)	5 (2.58)	20 (3.64)		5 (2.81)	4 (2.25)	
Nx, no. (%)	38 (5.11)	15 (7.73)	23 (4.19)		11 (6.18)	11 (6.18)	
**Radiotherapy**				0.21			1.00
Yes, no. (%)	433 (58.28)	121 (62.37)	312 (56.83)		65 (36.52)	66 (37.08)	
No/Unknown, no. (%)	310 (41.72)	73 (37.63)	237 (43.17)		113 (63.48)	112 (62.92)	
**Chemotherapy**				0.41			0.78
Yes, no. (%)	568 (76.45)	153 (78.87)	415 (75.59)		144 (80.90)	147 (82.58)	
No/Unknown, no. (%)	175 (23.55)	41 (21.13)	134 (24.41)		34 (19.10)	31 (17.42)	
**Biopsy of nasopharynx^ b^**				0.51			1.00
Yes, no. (%)	375 (50.47)	91 (46.91)	284 (51.73)		85 (47.75)	86 (48.31)	
No, no. (%)	350 (47.11)	98 (50.52)	252 (45.9)		93 (52.25)	92 (51.69)	
**Biopsy of neck^ b^**							0.91
Yes, no. (%)	228 (30.69)	65 (33.51)	163 (29.69)	0.61	59 (33.15)	57 (32.02)	
No, no. (%)	500 (67.29)	125 (64.43)	375 (68.31)		119 (66.85)	121 (67.98)	

^a^ Chi-square statistic for nominal variables and Student's t-test for continuous variables; ^b^ some data were missing (7 cases in race, 16 cases in T stage, 16 cases in N stage, 18 cases in biopsy of nasopharynx and 15 cases in biopsy of neck). Abbreviations: UNKC: Undifferentiated nonkeratinizing carcinoma; DNKC: Differentiated nonkeratinizing carcinoma; KSCC: Keratinizing squamous cell carcinoma.

**Table 2:** Univariate analysis identifying prognostic factors for OS and CSS in the well-balanced cohort

Characteristics	Overall survival		Cancer specific survival	
	HR (95%CI)	*P* value	HR (95% CI)	*P* value
**Age**				
≤60 yrs	Ref		Ref	
>60 yrs	1.66 (1.29-2.13)	<0.01	1.36 (1.02-1.81)	0.03
**Sex**				
Female	Ref		Ref	
Male	1.34 (1.00-1.81)	0.05	1.39 (0.99-1.96)	0.06
**Year of diagnosis**				
<2010	Ref		Ref	
≥2010	1.02 (0.79-1.32)	0.89	1.00 (0.75-1.34)	0.99
**Race**				
Asian or Pacific Islander	Ref		Ref	
American Indian/Alaska Native/black	1.60 (1.11-2.30)	0.01	1.55 (1.03-2.35)	0.04
White	1.14 (0.87-1.51)	0.33	1.14 (0.83-1.55)	0.41
**Histology type**				
UNKC	Ref		Ref	
DNKC	1.01 (0.65-1.56)	0.96	1.15 (0.71-1.88)	0.57
KSC	1.73 (1.18-2.53)	0.01	1.88 (1.22-2.90)	<0.01
Other	1.34 (0.90-1.99)	0.14	1.24 (0.78-1.96)	0.36
**T stage**				
T0-2	Ref		Ref	
T3-4	1.29 (0.98-1.70)	0.07	1.33 (0.98-1.82)	0.07
Tx	1.53 (1.08-2.15)	0.02	1.41 (0.95-2.10)	0.09
**N stage**				
N0	Ref		Ref	
N1-2	0.78 (0.55-1.09)	0.15	0.98 (0.65-1.47)	0.92
N3/Nx	1.09 (0.75-1.58)	0.66	1.17 (0.75-1.82)	0.50
Radiotherapy	0.59 (0.46-0.76)	<0.01	0.58 (0.44-0.77)	<0.01
Chemotherapy	0.28 (0.21-0.37)	<0.01	0.30 (0.22-0.43)	<0.01
Biopsy of nasopharynx	1.02 (0.79-1.30)	0.89	0.99 (0.75-1.31)	0.95
Biopsy of neck	0.98 (0.75-1.27)	0.85	1.09 (0.82-1.46)	0.56
Biopsy of distant metastasis	1.11 (0.87-1.42)	0.41	1.14 (0.86-1.52)	0.35

Abbreviations: UNKC: Undifferentiated nonkeratinizing carcinoma; DNKC: Differentiated nonkeratinizing carcinoma; KSCC: Keratinizing squamous cell carcinoma.

**Table 3:** Multivariate analysis assessing independent prognostic factors for OS and CSS in the entire cohort

Characteristics	Overall survival		Cancer specific survival	
	HR (95% CI)	*P* value	HR (95% CI)	*P* value
**Age**				
≤60 yrs	Ref		Ref	
>60 yrs	1.50 (1.24-1.81)	<0.01	1.27 (1.02-1.57)	0.03
**Race**				
Asian or Pacific Islander	Ref		Ref	
American Indian/Alaska Native/black	1.24 (0.97-1.59)	0.08	1.16 (0.88-1.53)	0.29
White	1.15 (0.94-1.40)	0.17	1.10 (0.88-1.37)	0.4
**Histology**				
UNKC	Ref		Ref	
DNKC	0.96 (0.70-1.33)	0.82	1.14 (0.80-1.64)	0.46
KSC	1.73 (1.31-2.28)	<0.01	1.90 (1.38-2.60)	<0.01
Other	1.32 (1.00-1.75)	0.05	1.43 (1.04-1.98)	0.03
**T stage**				
T0-2	Ref		Ref	
T3-4	1.38 (1.13-1.67)	<0.01	1.47 (1.18-1.83)	<0.01
Tx	1.04 (0.79-1.37)	0.78	1.08 (0.79-1.47)	0.63
**N stage**				
N0	Ref		Ref	
N1-2	1.38 (1.05-1.80)	0.02	1.57 (1.14-2.16)	0.01
N3/NX	1.39 (1.03-1.86)	0.03	1.54 (1.09-2.17)	0.01
Chemotherapy	0.40 (0.32-0.49)	<0.01	0.45 (0.35-0.57)	<0.01
Radiotherapy	0.66 (0.54-0.80)	<0.01	0.62 (0.50-0.77)	<0.01
Biopsy of distant metastasis	1.03 (0.84-1.25)	0.80	1.07 (0.86-1.34)	0.54

Abbreviations: UNKC: Undifferentiated nonkeratinizing carcinoma; DNKC: Differentiated nonkeratinizing carcinoma; KSCC: Keratinizing squamous cell carcinoma.

## References

[1] Browse the SEER Cancer Statistics Review (CSR) 1975-2012. https://seer.cancer.gov/archive/csr/1975_2012/browse_csr.php?sectionSEL=20&pageSEL=sect_20_table.09

[2] Ferlay J EM, Lam F, Colombet M, Mery L, Piñeros M, Znaor A, Soerjomataram I, Bray F (2018). Global Cancer Observatory: Cancer Today. Lyon, France: International Agency for Research on Cancer. Available from: https://gco.iarc.fr/today, accessed [Jul.11 2020].

[3] Lee AWM, Ng WT, Chan LK, Chan OSH, Hung WM, Chan CC (2012). The strength/weakness of the AJCC/UICC staging system (7th edition) for nasopharyngeal cancer and suggestions for future improvement. Oral oncology.

[4] Dickson RI (1981). Nasopharyngeal carcinoma: an evaluation of 209 patients. The Laryngoscope.

[5] Cao SM, Yang Q, Guo L, Mai HQ, Mo HY, Cao KJ (2017). Neoadjuvant chemotherapy followed by concurrent chemoradiotherapy versus concurrent chemoradiotherapy alone in locoregionally advanced nasopharyngeal carcinoma: A phase III multicentre randomised controlled trial. Eur J Cancer.

[6] Lee AW, Tung SY, Chua DT, Ngan RK, Chappell R, Tung R (2010). Randomized trial of radiotherapy plus concurrent-adjuvant chemotherapy vs radiotherapy alone for regionally advanced nasopharyngeal carcinoma. J Natl Cancer Inst.

[7] Sun Y, Li W-F, Chen N-Y, Zhang N, Hu G-Q, Xie F-Y (2016). Induction chemotherapy plus concurrent chemoradiotherapy versus concurrent chemoradiotherapy alone in locoregionally advanced nasopharyngeal carcinoma: a phase 3, multicentre, randomised controlled trial. The Lancet Oncology.

[8] Lee V, Kwong D, Leung TW, Lam KO, Tong CC, Lee A (2017). Palliative systemic therapy for recurrent or metastatic nasopharyngeal carcinoma - How far have we achieved?. Critical reviews in oncology/hematology.

[9] Chen MY, Jiang R, Guo L, Zou X, Liu Q, Sun R (2013). Locoregional radiotherapy in patients with distant metastases of nasopharyngeal carcinoma at diagnosis. Chinese journal of cancer.

[10] Yeh SA, Tang Y, Lui CC, Huang EY (2006). Treatment outcomes of patients with AJCC stage IVC nasopharyngeal carcinoma: benefits of primary radiotherapy. Japanese journal of clinical oncology.

[11] Tang LQ, Chen QY, Fan W, Liu H, Zhang L, Guo L (2013). Prospective study of tailoring whole-body dual-modality [18F]fluorodeoxyglucose positron emission tomography/computed tomography with plasma Epstein-Barr virus DNA for detecting distant metastasis in endemic nasopharyngeal carcinoma at initial staging. Journal of clinical oncology: official journal of the American Society of Clinical Oncology.

[12] Hsu C, Lee SH, Ejadi S, Even C, Cohen RB, Le Tourneau C (2017). Safety and Antitumor Activity of Pembrolizumab in Patients With Programmed Death-Ligand 1-Positive Nasopharyngeal Carcinoma: Results of the KEYNOTE-028 Study. Journal of clinical oncology: official journal of the American Society of Clinical Oncology.

[13] Faltas BM, Prandi D, Tagawa ST, Molina AM, Nanus DM, Sternberg C (2016). Clonal evolution of chemotherapy-resistant urothelial carcinoma. Nature genetics.

[14] Ullah I, Karthik GM, Alkodsi A, Kjц╓llquist U, Stц╔lhammar G, Lц╤vrot J (2018). Evolutionary history of metastatic breast cancer reveals minimal seeding from axillary lymph nodes. The Journal of clinical investigation.

[15] Yates LR, Knappskog S, Wedge D, Farmery JHR, Gonzalez S, Martincorena I (2017). Genomic Evolution of Breast Cancer Metastasis and Relapse. Cancer cell.

[16] Riggins RS, Ketcham AS (1965). EFFECT OF INCISIONAL BIOPSY ON THE DEVELOPMENT OF EXPERIMENTAL TUMOR METASTASES. The Journal of surgical research.

[17] Heimbach JK, Sanchez W, Rosen CB, Gores GJ (2011). Trans-peritoneal fine needle aspiration biopsy of hilar cholangiocarcinoma is associated with disease dissemination. HPB: the official journal of the International Hepato Pancreato Biliary Association.

[18] Yang XL, Wang Y, Bao Y, Liang SB, He SS, Chen DM (2018). Additional Cervical Lymph Node Biopsy is Not a Significant Prognostic Factor for Nasopharyngeal Carcinoma in the Intensity-Modulated Radiation Therapy Era: A Propensity Score-matched Analysis from an Epidemic Area. Journal of Cancer.

[19] Lv JW, Zhou GQ, Chen YP, Tang LL, Mao YP, Chen L (2017). Refining the Role of Lymph Node Biopsy in Survival for Patients with Nasopharyngeal Carcinoma: Population-Based Study from the Surveillance Epidemiology and End-Results Registry. Annals of surgical oncology.

[20] National Comprehensive Cancer Network. NCCN Guidelines: Head and neck cancers; Version1.2015. Available at: http://www.nccn.org/

[21] Surveillance, Epidemiology, and End Results (SEER) Program (www.seer.cancer.gov) SEER*Stat Database: Incidence - SEER Research Data, 18 Registries, Nov 2018 Sub (1975-2016) - Linked To County Attributes - Time Dependent (1990-2016) Income/Rurality, 1969-2016 Counties, National Cancer Institute, DCCPS, Surveillance Research Program, released April 2019, based on the November 2018 submission

[22] World Health Organization (2013). International classification of diseases for oncology (ICD-O) - 3rd edition, 1st revision, 3rd ed. https://apps.who.int/iris/handle/10665/96612.

[23] Austin PC (2010). Statistical criteria for selecting the optimal number of untreated subjects matched to each treated subject when using many-to-one matching on the propensity score. American journal of epidemiology.

[24] Kara M, Alver G, Sak SD, Kavukц╖u S (2001). Implantation metastasis caused by fine needle aspiration biopsy following curative resection of stage IB non-small cell lung cancer. European journal of cardio-thoracic surgery: official journal of the European Association for Cardio-thoracic Surgery.

[25] Merker JD, Oxnard GR, Compton C, Diehn M, Hurley P, Lazar AJ (2018). Circulating Tumor DNA Analysis in Patients With Cancer: American Society of Clinical Oncology and College of American Pathologists Joint Review. Journal of clinical oncology: official journal of the American Society of Clinical Oncology.

[26] Xu RH, Wei W, Krawczyk M, Wang W, Luo H, Flagg K (2017). Circulating tumour DNA methylation markers for diagnosis and prognosis of hepatocellular carcinoma. Nature materials.

[27] Simmons C, Miller N, Geddie W, Gianfelice D, Oldfield M, Dranitsaris G (2009). Does confirmatory tumor biopsy alter the management of breast cancer patients with distant metastases?. Annals of oncology: official journal of the European Society for Medical Oncology.

[28] Tsang J, Lee VH, Kwong DL (2014). Novel therapy for nasopharyngeal carcinoma - where are we. Oral oncology.

[29] Hui EP, Ma BB, King AD, Mo F, Chan SL, Kam MK (2011). Hemorrhagic complications in a phase II study of sunitinib in patients of nasopharyngeal carcinoma who has previously received high-dose radiation. Annals of oncology: official journal of the European Society for Medical Oncology.

[30] Hui EP, Ma BBY, Loong HHF, Mo F, Li L, King AD (2018). Efficacy, Safety, and Pharmacokinetics of Axitinib in Nasopharyngeal Carcinoma: A Preclinical and Phase II Correlative Study. Clinical cancer research: an official journal of the American Association for Cancer Research.

[31] Ma BBY, Lim WT, Goh BC, Hui EP, Lo KW, Pettinger A (2018). Antitumor Activity of Nivolumab in Recurrent and Metastatic Nasopharyngeal Carcinoma: An International, Multicenter Study of the Mayo Clinic Phase 2 Consortium (NCI-9742). Journal of clinical oncology: official journal of the American Society of Clinical Oncology.

[32] Qu Q, Zong Y, Fei XC, Chen XS, Xu C, Lou GY (2014). The importance of biopsy in clinically diagnosed metastatic lesions in patients with breast cancer. World journal of surgical oncology.

